# Nomogram for anemia risk prediction and validation in sepsis patients

**DOI:** 10.1016/j.isci.2025.114483

**Published:** 2025-12-18

**Authors:** Songwei Li, Yahui Gao, Tianyu Xin, Jianying Guo, Liwei Pang, Li Yan, Fei She, Xiaohong Li, Shuangqing Liu

**Affiliations:** 1 Institute of Hematology, The Fifth Medical Center of the Chinese PLA General Hospital, Beijing 100071, China; 2 Department of Emergency, The Sixth Medical Center of the Chinese PLA General Hospital, Beijing 100048, China; 3 Department of Surgical Intensive Care Unit, The Fourth Medical Center of the Chinese PLA General Hospital, Beijing 100048, China; 4 Department of Anesthesiology, The Fourth Medical Center of the Chinese PLA General Hospital, Beijing 100048, China; 5 Department of Emergency, The Fourth Medical Center of the Chinese PLA General Hospital, Beijing 100048, China

**Keywords:** health sciences

## Abstract

Anemia is a common sepsis complication with poor long-term outcomes, and accurate risk stratification tools are critical for clinical management. We conducted a multicenter retrospective observational study to develop and validate a nomogram for anemia in Chinese Han sepsis patients, enrolling 723 eligible patients from two tertiary hospitals. Lasso regression for parameter screening and binary logistic regression identified age, gender, APACHE Ⅱ scores, albumin (Alb), procalcitonin (PCT), and cholinesterase (ChE) as independent predictors. Based on multifactorial results of the binary logistic regression analysis in the training cohort, a nomogram was established to predict the anemia risk in sepsis patients. The nomogram exhibited excellent discriminative performance (AUC: 0.911 [95% CI, 0.880–0.936] in the training set, 0.896 [95% CI, 0.843–0.936] internally, 0.876 [95% CI, 0.801–0.931] externally) and good calibration. These findings provide a highly accurate nomogram for anemia risk prediction in this population, offering a reliable tool for clinical decision-making.

## Highlights

• First validated nomogram for anemia risk prediction in Chinese Han sepsis patients
• Key independent predictors of anemia occurrence in sepsis are identified
• The model shows excellent discriminative ability and robust calibration
• Enables early anemia risk stratification and targeted interventions to improve outcomes

## Graphical abstract

**Figure:**
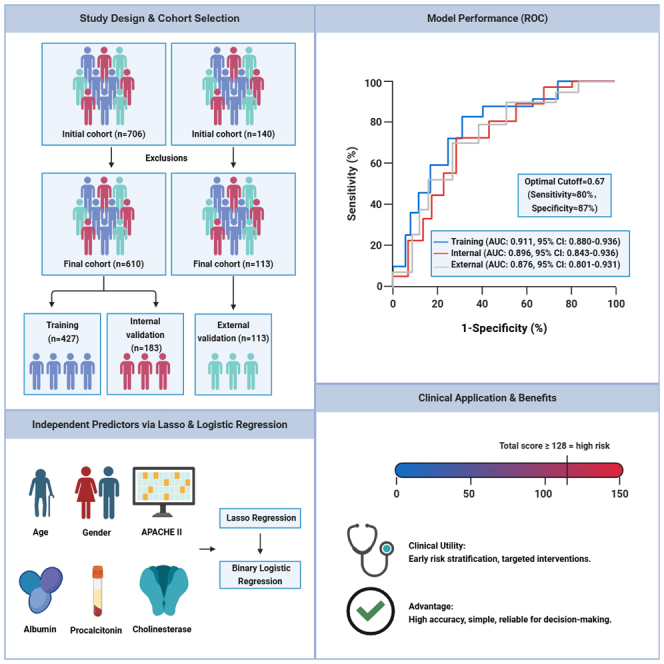

## Introduction

Sepsis is one of the leading causes of mortality and morbidity among critically ill patients worldwide, which affects more than 750,000 individuals annually and caused 200,000 deaths per year in the United States.^1^^,^^2^ In addition to high mortality and morbidity, sepsis is a huge burden across all economic regions.^3^ Despite tremendous advances in critical care, the treatment of sepsis is still extremely difficult, and the occurrence of various adverse complications has a profound impact on the prognosis of septic patients.

As a common complication of critical illness, anemia is usually manifested as fatigue, shortness of breath, and cardiac complications.^4^ Of note, anemia is also regarded as a common feature of sepsis, and the etiology of this anemia is multifactorial and includes fluid-related hemodilution, iatrogenic blood loss, decreased red blood cell production, depression of serum iron levels and erythropoietin production, and increased red blood cell destruction.^5^^,^^6^ Furthermore, sepsis-related chronic critical illness is a condition characterized by the pathophysiology of persistent inflammation, immunosuppression, and protein catabolism leading to poor outcomes. Decreased hemoglobin levels have been demonstrated to be associated with persistent inflammation in septic patients.^7^^,^^8^^,^^9^ Thus, the hemoglobin concentration may change with the severity of illness-associated parameters in patients with sepsis. Meanwhile, most patients in intensive care units (ICU) suffer from anemia as a result of nutritional deficiencies, blood losses, inflammation, or another disease process.^7^ Numerous studies have reported that malnutrition was a contributing factor in the development of anemia.^7^^,^^10^^,^^11^^,^^12^

Theoretically, it is conceivable that throughout the course of sepsis the severity of illness and state of nutrition may influence the hemoglobin concentration. We hypothesized that during the first hours of hospital admission, the disease severity and nutritional status of septic patients have a greater effect on the hemoglobin concentration and may be the leading cause for a reduction in hemoglobin levels. However, few studies have investigated the role of these physiological parameters above mentioned in predicting the risk of anemia in patients with sepsis. It is imperative to accurately forecast the likelihood of anemia development in septic patients. Blood transfusion may increase the risk of complications such as infection and immunosuppression. Predicting the risk of anemia can help physicians balance blood transfusion indications, avoid unnecessary blood transfusions by supplementing hematopoietic materials (such as iron agents and erythropoietin) or timely controlling inflammation, and maintain circulatory stability through other means (such as improving tissue oxygen supply and correcting coagulation abnormalities). Hence, we aimed to describe the relationship between the reduction in hemoglobin concentration and pathophysiological indicators in order to develop a nomograph model of anemia risk for Chinese Han patients with sepsis.

## Results

### Baseline clinical characteristics

**Figure 1:**
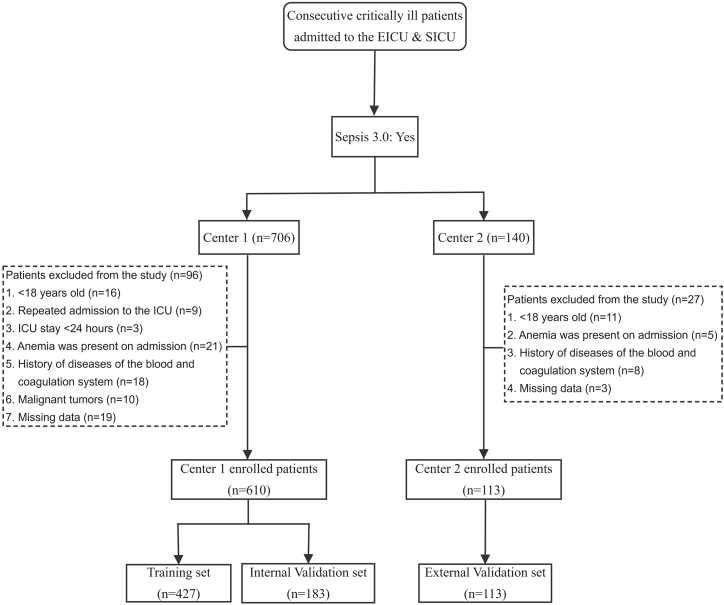
The flowchart for population selection

During the study period, a total of 723 patients with sepsis who meet the criteria were enrolled in this study (Figure 1). Among them, 290 patients (67.9%) developed anemia for the training cohort, 108 patients (59.0%) suffered from anemia for the internal validation set, and 68 patients (60.2%) were diagnosed with anemia for the external validation set. The baseline information of eligible patients in the training set is displayed in Table 1. At first presentation to the ICU, there were no significant differences in the gender, body temperature, systolic blood pressure (SBP), heart rate, comorbidities, drug usage, and personal life history between both groups (*p* > 0.05). The septic patients developing anemia were significantly older than septic patients in the non-anemia group (*p* < 0.001). Additionally, compared to septic patients in the non-anemia group, those in the anemia group were more likely to occur in patients with lower body mass index (BMI) (*p* < 0.05).

**Table 1:** Baseline characteristics of the 427 patients with sepsis in the training set

Variables	Anemia, *n* = 290	Non-anemia, *n* = 137	*p* value
**Demographics and vital signs**			

Gender (male; no. [%])	154 (53.1)	70 (51.1)	0.698
Age (years)	63.21 ± 11.24	53.48 ± 13.47	<0.001
BMI (kg/m^2^)	21.93 ± 2.56^a^	22.75 ± 2.95	0.004
Body temperature (°C)	37.67 ± 0.86	37.81 ± 0.90	0.110
SBP (mmHg)	123.07 ± 16.23	123.28 ± 15.21	0.896
Heart rate (bpm)	77.61 ± 11.15	78.54 ± 10.35	0.411

**Comorbidities (no. [%])**			

Hypertension	56 (19.3)	35 (25.5)	0.142
Diabetes mellitus	49 (17.0)^a^	17 (12.4)	0.221
IHD	26 (9.0)	10 (7.3)	0.563
COPD	45 (15.5)	20 (14.8)^a^	0.851

**Drug usage (no. [%])**			

Antiplatelet drug	47 (16.2)	26 (19.0)	0.478
Proton pump inhibitors	18 (6.6)^a^	6 (4.4)	0.363
Statins	88 (31.3)^a^	37 (29.1)^a^	0.658
ACEI/ARB	126 (46.3)^a^	71 (51.8)	0.293

**Personal life history (no. [%])**			

Cigarettes	69 (23.8)	43 (31.4)	0.096
Alcohol	65 (23.5)^a^	37 (27.0)	0.431

Abbreviations: SBP, systolic blood pressure; bpm, beats per minute; IHD, ischemic heart disease; COPD, chronic obstructive pulmonary disease; ACEI/ARB, angiotensin-converting enzyme inhibitors/angiotensin II receptor blockers. Data are expressed as the mean ± SD, or number (%). A *p* value < 0.05 indicates statistical significance.

a Containing partial missing data.

### Comparison of severity scores and relevant studied biomarkers

**Figure 2:**
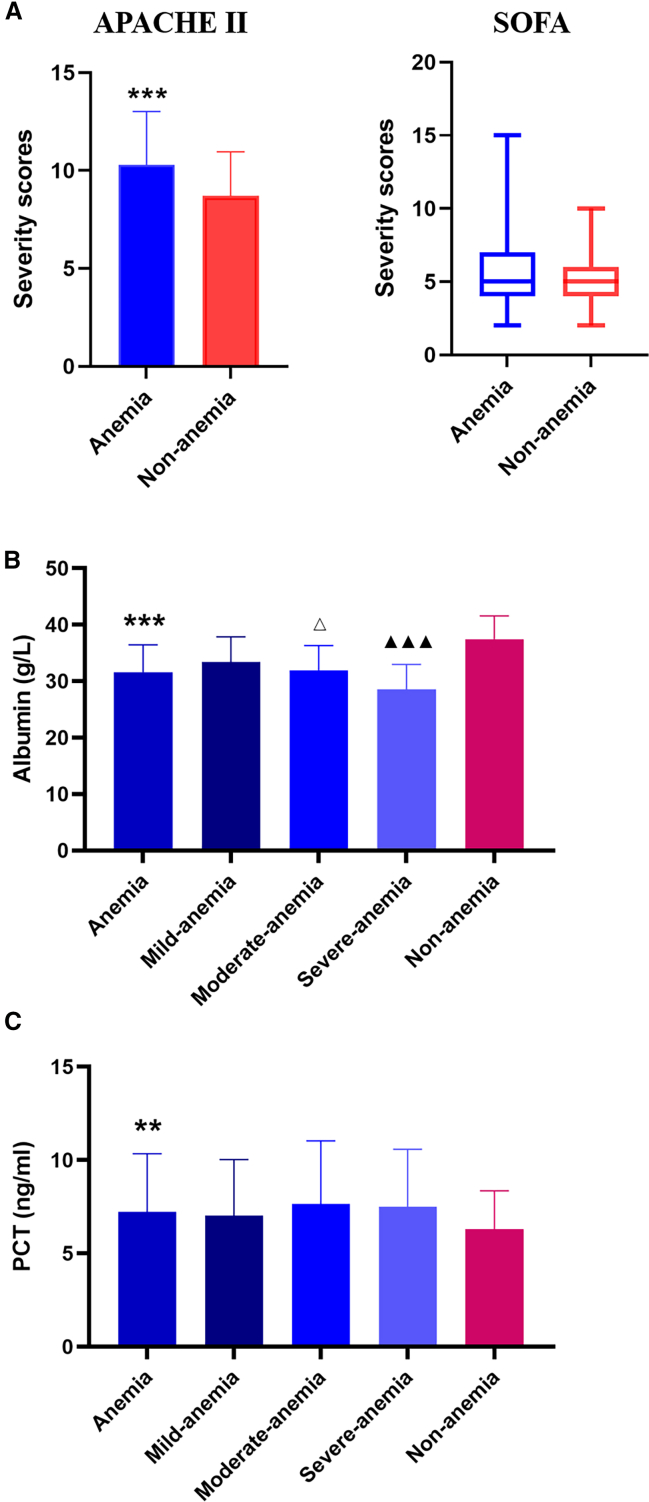
The severity scores, albumin, and PCT levels in both groups in the training set All the data including (A), albumin (B), and PCT (C) levels were obtained based on the clinical characteristics and laboratory examinations of septic patients. (A) Comparison of severity scores between the anemia group (*n* = 290) and the non-anemia group (*n* = 137). (APACHE Ⅱ, Mean ± SD, *t* = 5.941, ∗∗∗*p* < 0.001; SOFA, IQR, *Z* = −0.876). (B) Comparison of albumin between the anemia group and the non-anemia group, as well as among the subgroups of anemia (mild, *n* = 113; moderate, *n* = 100; severe, *n* = 77). (Mean ± SD, *t* = −12.217, ∗∗∗*p* < 0.001, compared with the non-anemia group; Mean ± SD, *F* = 27.447, compared with the mild anemia subgroup, ^Δ^*p* < 0.05; compared with the moderate anemia subgroup, ^▲▲▲^*p* < 0.001, one-way ANOVA). (C) Comparison of PCT between the anemia group and the non-anemia group, as well as among the subgroups of anemia (mild, *n* = 113; moderate, *n* = 100; severe, *n* = 77). (Mean ± SD, *t* = 2.734, ∗∗*p* < 0.01, compared with the non-anemia group; Mean ± SD, *F* = 1.124, compared with the mild anemia subgroup, one-way ANOVA).

To evaluate the impact of severity of illness on the anemia occurrence in septic patients, we chose the Acute Physiology and Chronic Health Evaluation (APACHE) and Sequential Organ Failure Assessment (SOFA) scores as observation targets. As shown in Figure 2A, it was found that the APACHE II scores were much higher in the anemia group compared with the non-anemia group (*p* < 0.001). Whereas, there was no significant difference in the SOFA scores between the anemia and non-anemia group (*p* > 0.05), and the detailed data are displayed in Table S1.

The albumins (Alb) are the most abundant proteins in plasma of healthy human and are being used as indicators of nutritional status and hepatic function based on the assumption that plasma levels reflect the rate of albumin synthesis.^13^ In this study, we found that the albumin levels were markedly lower in the anemia group than that in the non-anemia group (Figure 2B; *p* < 0.05). Furthermore, we separated the patients in the anemia group into three subgroups according to hemoglobin concentrations. We observed that the albumin concentration was the lowest in the severe anemia group, and highest in the mild anemia group (Table S2). In addition, lower procalcitonin (PCT) level was found in the non-anemia group (Figure 2C and Table S2; *p* < 0.05); however, no significant difference was found among the three subgroups (Figure 2C and Table S2; *p* > 0.05).

### Predictors of the anemia risk in septic patients

**Figure 3:**
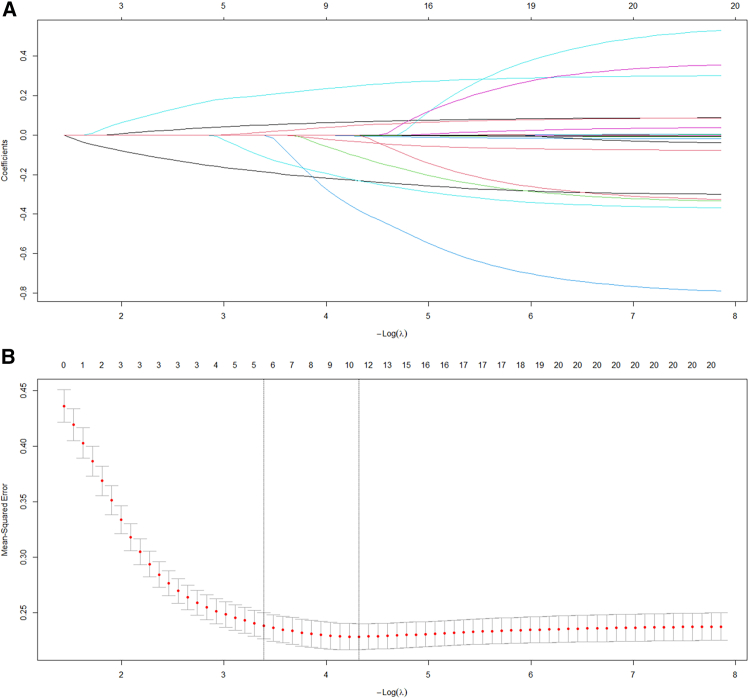
Screening of variables based on Lasso regression (A) The variation characteristics of the coefficient of variables. (B) The process of selecting the optimal value of parameter λ in the Lasso regression model via cross-validation.

Lasso regression was employed to screen potential parameters from 20 candidate parameters, and the variation characteristics of the coefficient of these variables were shown in Figure 3A. The 10-fold cross-validation approach was utilized for iterative analysis, and a model with excellent performance but minimum number of variables was obtained when *λ* reached 0.013 (Log *λ* = −1.876) (Figure 3B). The screened parameters included age, BMI, heart rate, APACHE II scores, Alb, PCT, C-reactive protein (CRP), gender, cholinesterase (ChE), interleukin (IL)-6, lactate, and statins usage.

In the binary logistic regression model, we performed multivariate analysis of 12 parameters including demographic characteristics, severity scores, and laboratory findings in the training set. As revealed in Table 2, the age, gender, APACHE Ⅱ scores, Alb, PCT, and ChE levels were associated with anemia development independently in septic patients (*p* < 0.05).

**Table 2:** Multivariate binary logistic regression to predict anemia based on Lasso regression

Variables	β	SE	Wald	*p* value	OR	95% CI
Age	0.090	0.014	42.460	<0.001	1.094	1.065–1.124
APACHE Ⅱ	0.309	0.074	17.596	<0.001	1.363	1.179–1.574
Alb	−0.292	0.039	55.108	<0.001	0.746	0.691–0.806
PCT	0.084	0.034	6.281	0.012	1.088	1.019–1.162
ChE	−0.361	0.166	4.737	0.030	0.697	0.503–0.965
Gender	0.707	0.333	4.516	0.034	2.028	1.056–3.891
BMI	−0.091	0.057	2.513	0.113	0.913	0.817–1.022
Heart rate	−0.017	0.016	1.199	0.273	0.983	0.954–1.014
CRP	−0.006	0.006	1.016	0.313	0.994	0.982–1.006
IL-6	−0.004	0.005	0.620	0.431	0.996	0.987–1.006
Lactate	−0.314	0.187	2.839	0.092	0.730	0.507–1.053
Statins usage	0.296	0.354	0.700	0.403	1.345	0.672–2.693

OR, odds ratio; CI, confidence intervals. *p* value < 0.05 indicates statistical significance.

### Construction of nomogram

**Figure 4:**
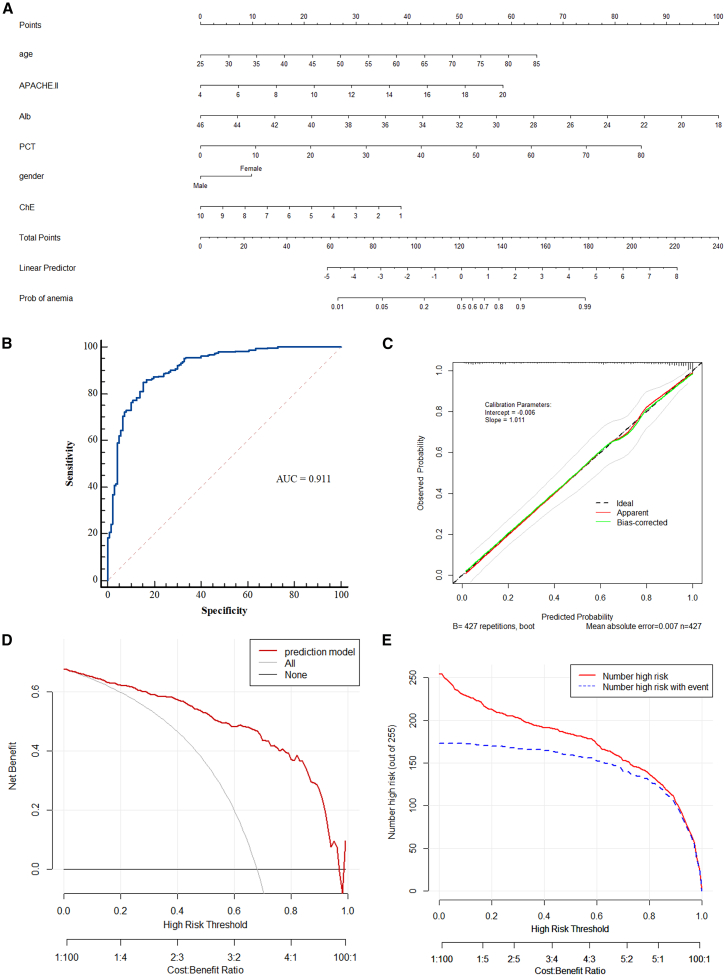
The nomogram for predicting the anemia risk in ICU patients with sepsis (A) The nomogram is used by summing all the points for each variable on the scale. The total score projected on the bottom scale indicates the possibility of anemia in patients with sepsis. (B) ROC curves of the training set (the cutoff value is 0.67). (C) Calibration curves predict the possibility of anemia occurrence in the training set. (D) Decision curve analysis for the training set. (E) Clinical impact curve analysis for the training set.

Based on the multifactorial results of the binary logistic regression analysis in the training cohort, a nomogram was established to predict the anemia risk in septic patients (Figure 4A). A straight line perpendicular to the points line was drawn to obtain the scores corresponding to different features of each variable, and the points of features of each variable were added up. Then, the sum was located on the total-point scale axis and draw a straight line perpendicular to the risk axis, in order to determine the probability of anemia in septic patients.

### Evaluation and validation of the prediction model

**Figure 5:**
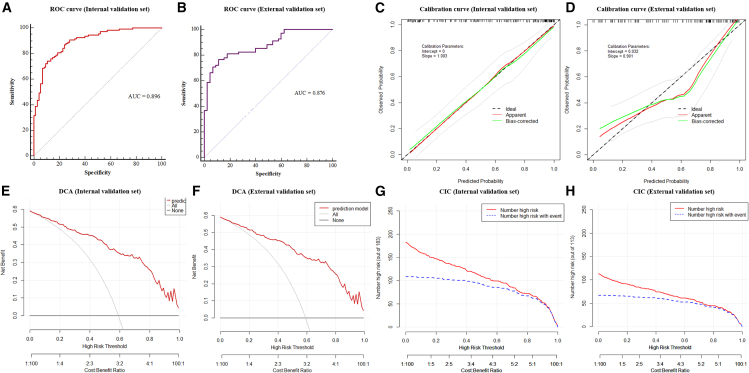
Internal and external validation for predictive performance of the nomogram (A) ROC curve for the internal validation set. (B) ROC curve for the external validation set. Calibration curve for predicting anemia development in the internal validation set (C) and external validation set (D). Decision curve analysis for the internal validation set (E) and external validation set (F). Clinical impact curve analysis for the internal validation set (G) and external validation set (H).

The nomogram exhibited a robust prediction capability in both the training and internal validation sets. The area under the receiver operating characteristic curve (AUC) of the nomogram in the training cohort was 0.911 (95% confidence interval [CI], 0.880–0.936), and 0.896 (95% CI, 0.843–0.936) in the internal validation cohort (Figures 4B and 5A and Table 3). The nomogram achieved a highest AUC in the training cohort, with a specificity of 84.67% and a sensitivity of 84.83%. The calibration curves showed a good concordance between the bias-corrected prediction and ideal reference lines in the training and internal validation cohorts (Figures 4C and 5C). The Hosmer-Lemeshow Test was utilized to evaluate the overall goodness of fit of the nomogram. The test returned a chi-square value of 14.262 and a *p* value of 0.075, indicating that the model fits the data adequately at the conventional significance level (*p* > 0.05). Additionally, decision curve analysis (DCA) was performed to assess the predictive efficiency of the nomogram, which indicated that the nomogram may provide clinical benefit to patients with sepsis, compared to no treatment or an all-treatment approach, for predicted probability thresholds ranging from 0% to 98% (the training cohort) and 15%–98% (the internal validation cohort) (Figures 4D and 5E). At last, clinical impact curve (CIC) was plotted to evaluate the clinical applicability of risk prediction nomogram (Figures 4E and 5G), which visually showed that the nomogram had a superior overall net benefit within the wide and practical ranges of threshold probabilities and impacted patient outcomes in the training and internal validation cohorts.

**Table 3:** Receiver operating characteristic analysis of factors independently predicting anemia risk in septic patients

Cohort	AUC	95% CI	Sensitivity	Specificity	Youden index
Training cohort	0.911	0.880–0.936	84.83%	84.67%	0.6950
Internal validation cohort	0.896	0.843–0.936	74.07%	89.33%	0.6341
External validation cohort	0.876	0.801–0.931	76.47%	88.89%	0.6536

Cutoff value = 0.67.

Importantly, external validation was performed using a dataset of 113 septic patients at the Sixth Medical Center of the Chinese PLA General Hospital. Compared with patients from Center 1, those from Center 2 were older, had a higher BMI, lower baseline blood pressure, and higher proportions of both diabetes mellitus and antiplatelet drug use (Table 4) (*p* < 0.05). Although there are differences in the demographic characteristics between the two centers, the nomogram continued to demonstrate excellent prediction accuracy with an AUC of 0.876 (95% CI, 0.801–0.931) (Figure 5B and Table 3). The calibration curve also exhibited good consistency between the predicted and observed risk of anemia in the population of external validation set (Figure 5D). Using the DCA, we found that at a threshold probability of 5%–98%, the net benefit of classifying patients at high risk of anemia using the probability predicted by nomogram would be higher than that of treating all or nothing (Figure 5F). Moreover, the CIC of external validation set shows that the nomogram also has excellent performance in potential clinical utility (Figure 5H).

**Table 4:** Demographic characteristics of septic patients between two centers

Variables	Center #1 (*n* = 610)	Center #2 (*n* = 113)	*p* value
**Demographics and vital signs**			

Gender (male; no. [%])	335 (54.9)	56 (49.6)	0.294
Age, (years)	58.02 ± 13.17	62.09 ± 8.55	0.002
BMI (kg/m^2^)	22.20 ± 2.70^a^	23.09 ± 2.64	0.001
Body temperature (°C)	37.82 ± 0.89	37.96 ± 0.84	0.118
SBP (mmHg)	118.86 ± 17.49	107.19 ± 13.68	<0.001
Heart rate (bpm)	85.08 ± 16.41	87.94 ± 14.08	0.083

**Comorbidities (no. [%])**			

Hypertension	119 (19.5)	30 (26.5)	0.089
Diabetes mellitus	91 (15.0)^a^	29 (25.7)	0.005
IHD	54 (8.9)	11 (10.7)^a^	0.551
COPD	93 (13.7)^a^	9 (8.0)	0.096

**Drug usage (no. [%])**			

Antiplatelet drug	87 (14.3)	26 (23)	0.019
Proton pump inhibitors	29 (4.9)^a^	6 (5.4)^a^	0.825
Statins	150 (25.6)^a^	31 (27.4)	0.683
ACEI/ARB	237 (40.3)^a^	41 (36.3)	0.423

**Personal life history (no. [%])**			

Cigarettes	137 (22.4)	23 (20.4)	0.621
Alcohol	136 (22.9)^a^	20 (17.7)	0.222

a Containing partial missing data. A *p* value < 0.05 indicates statistical significance.

To achieve better prediction performance of the model, we identified the optimal balance point on the receiver operator characteristic curve (ROC) curve and used it as the threshold. Septic patients with a possibility of anemia greater than 0.67 were defined as a population with a high risk of developing anemia, and the corresponding score on the nomogram prediction model was 128 (Table 3 and Figure 4A). Collectively, these results suggest that this prediction model possesses significant predictive value.

## Discussion

Over the past few decades, sepsis is always the focus of medical attention and research.^14^ Anemia, as a common complication in critically ill patients in the ICU, is known to persist months after discharge and is associated with reduced quality of life after hospitalization. It was reported that critically ill patients had a persistent anemia that was associated with chronic systemic inflammation and dreadful functional outcomes six months following sepsis.^8^ Therefore, early identification of the risk of anemia from sepsis is important for the timely and effective management and intervention, so as to improve outcomes of septic patients. The aim of this study was to evaluate the risk of anemia in Chinese Han patients with sepsis hospitalized in the ICU using a prediction model.

As is known, understanding sepsis-associated anemia’s varied and complex etiology is crucial for developing effective interventions that address causes of anemia and for monitoring anemia control programs. Chaparro et al.^10^ summarized risk factors most prevalent in low- and middle-income countries, including nutritional deficiencies, infection/inflammation, and genetic hemoglobin disorders. Hypoalbuminemia is common in hospitalized patients and is associated with poor clinical outcomes. Low serum albumin levels were initially considered to result from protein malnutrition or “kwashiorkor-like” malnutrition,^15^ although the relationship between serum albumin and nutritional status remains controversial. Many clinicians still believe that hypoalbuminemia is indicative of impaired nutritional status and reflects effects of nutrition.^16^^,^^17^ Eckart et al.^18^ conducted an observational and prospective study and reported that elevated parameters of inflammation and high nutritional risk were independently associated with hypoalbuminemia during acute illness, which implies that serum albumin could be affected by many different factors. In our retrospective study, we demonstrated that the albumin levels were significantly lower in the anemia group than in the non-anemia group (*p* < 0.05). These data indicated that serum albumin may serve as a potential predictive marker for anemia in sepsis and is not only considered a reliable nutritional marker in inflammatory states but also a marker for disease severity.

We also found that APACHE II scores were positively associated with criteria of sepsis associated anemia (*p* < 0.001). In clinical diagnosis and treatment, the APACHE II score is commonly used to evaluate the severity of the disease.^19^^,^^20^ With regard to the SOFA score, there was no significant difference between two groups. Similarly, there was no significant difference in CRP values between two groups. It was also noticed that the PCT values in the anemia group were obviously higher than those in the non-anemia group. However, PCT values in the anemia group failed to exhibit significant differences among there subgroups. As we know, PCT is one of the biomarkers that have been studied most frequently for the diagnostic or prognostic prediction value in sepsis.^1^^,^^21^^,^^22^^,^^23^^,^^24^ PCT levels have been used in the past to determine the severity of infection and sepsis in patients, but rarely used to predict the risk of anemia in sepsis. The mechanism by which PCT influences anemia is complicated and remains unclear. The inflammatory hypoferremia and iron-restricted erythropoiesis maybe played a major role in the context of inflammatory disease,^25^ which can be reflected by an increase in PCT levels.

Additionally, we investigated the parameters that independently predicted the risk of anemia in sepsis, which can provide clinicians with a useful tool for timely early intervention and care planning in patients with sepsis. Our data showed that the age, gender, APACHE Ⅱ scores, Alb, PCT, and ChE levels were independent predictors of anemia risk in Chinese Han septic patients by using multivariate logistic regression model (Table 2). Based on the above six independent risk factors, we developed a clinical prediction model, and display it with a nomogram (Figure 4A). The nomogram is simple, intuitive, and convenient for clinicians to use on prognostic prediction of disease,^26^ which can be applied in clinical practice. It is worth noting that, all the predictive indicators are routine clinical testing items with fast detection speeds and low prices. We found that advanced ages, high APACHE II scores, and low serum albumin levels are strongly associated with anemia, which are also associated with poor prognosis in critical ill patients.^27^^,^^28^^,^^29^^,^^30^ The predictive performance of this nomogram was evaluated by plotting the ROC curve, and the result indicates that the AUC was 0.911 (95% CI, 0.880–0.936). The calibration curve showed that the predicted probability of anemia occurrence of the nomograph model was consistent with the actual probability of anemia occurrence in sepsis. Furthermore, the DCA curve showed that the threshold probabilities of the samples are all between 15% and 98%, and the net benefit rate is > 0, which has clinical practical value, suggesting that the model has good clinical application value in predicting the anemia risk patients. Below this threshold, the predicted risk of the outcome is sufficiently low to render prophylactic interventions unwarranted; above it, the risk is so high that intervention becomes essentially mandatory, given the high likelihood of anemia developing without such measures. Importantly, the clinical impact curve showed that the prediction probabilities of our model were highly consistent with the actual occurrence, and the clinical prediction efficiency was high. Notably, the internal validation set and external validation set was used to validate the prediction model, and the results showed that our established prediction model has good prediction efficacy in the validation queue as well (Figures 4 and 5 and Table 3). Overall, the nomograph model has important predictive value for the anemia risk in Chinese Han patients with sepsis.

In summary, the present study demonstrated that the age, gender, APACHE Ⅱ scores, Alb, PCT, and ChE levels are associated with the suffering of anemia in sepsis among Chinese Han people. The nomogram model developed in this study was shown to have a good predictive ability as well as discrimination by internal and external validation and can provide a reference for early screening of septic patients with high risk of anemia. Nevertheless, further prospective studies are warranted to validate the stability, reliability, and repeatability of this prediction model.

### Limitations of the study

This study has several limitations. First, our work is a retrospective observational study, which could inevitably subject to data loss and potential data bias. Therefore, a prospective cohort study is needed to further confirm our results. Second, due to only septic patients in ICU were considered, we were unable to confirm whether this nomograph prediction model was applicable to patients with sepsis who were not admitted to the ICU. In our upcoming research, we plan to tackle these factors and conduct more thorough studies. Third, population selection bias may exist due to the single-ethnicity cohort, and the model’s performance in non-Han Chinese or other ethnic populations remains to be verified. Moreover, our study failed to conduct an external validation through public database data to assess the universality of the model for different ethnic groups. In the future, we plan to conduct an international multi-center study by incorporating data from septic patients of different ethnicities, in order to systematically verify the robustness of this model across different populations.

## Resource availability

### Lead contact

Further information and requests for resources and reagents should be directed to and will be fulfilled by the lead contact, Shuangqing Liu (shuangqliu@163.com).

### Materials availability

This study did not generate new unique reagents.

### Data and code availability

• The clinical data supporting the findings of this study have been publicly deposited in Mendeley Data, with the official DOI accession code: https://doi.org/10.17632/fp6xdds387.1 (Version 1, full access link: https://data.mendeley.com/datasets/fp6xdds387/1).
• The machine learning modeling code used in this study has been deposited in Mendeley Data alongside the clinical data, with the same DOI accession code https://doi.org/10.17632/fp6xdds387.1.
• Any additional information required is available upon reasonable request to the lead contact.

## Acknowledgments

This work was supported by the grant from the 10.13039/501100001809National Natural Science Foundation of China (no. 82372170). The authors would like to thank all the colleagues and contributed to this study, and we appreciate Dr. Yuxiao Liu from the First Medical Center of the 10.13039/501100009580Chinese PLA General Hospital for her exceptional contribution to this work.

## Author contributions

S.Q.L. and X.H.L. led the conceptualization of the study and provided supervision throughout the research process; T.Y.X. and J.Y.G. conducted investigation and data curation; S.Q.L. and S.W.L., together with Y.H.G., developed the methodology; S.W.L. and Y.H.G. drafted the original manuscript, while S.Q.L., S.W.L., Y.H.G., and X.H.L. participated in the review and editing of the paper; L.Y., L.W.P., and F.S. performed formal analysis, data interpretation, and validation of the study results. All authors read and approved the final manuscript.

## Declaration of interests

The authors declare no competing interests.

## STAR★Methods

### Key resources table

**Table:** 

REAGENT or RESOURCE	SOURCE	IDENTIFIER
**Biological samples**		

Blood samples of sepsis patients	The Fourth Medical Center of the Chinese PLA General Hospital; The Sixth Medical Center of the Chinese PLA General Hospital	N/A

**Deposited data**		

Clinical data	Mendeley Data (https://data.mendeley.com/datasets/fp6xdds387/1)	DOI: https://doi.org/10.17632/fp6xdds387.1
Machine learning model code	Mendeley Data (https://data.mendeley.com/datasets/fp6xdds387/1)	DOI: https://doi.org/10.17632/fp6xdds387.1

**Software and algorithms**		

Prism 9.5	GraphPad Software	https://www.graphpad.com
SPSS (v22.0)	IBM SPSS Statistics	https://www.ibm.com/products/spss-statistics
R (v4.5.1) and packages	CRAN/Bioconductor	Various
MedCalc (v11.4.40)	MedCalc Software Ltd	https://www.medcalc.com.cn

### Experimental model and study participant details

#### Study approval and ethical statement

Ethics Approval and Patient Consent Statement: This study received approval from the Independent Ethics Committee of the Fourth Medical Center of the General Hospital of Chinese People’s Liberation Army (2024KY0108-KS001). This study was conducted in accordance with the Declaration of Helsinki and relevant ethical regulations. Informed consent was waived due to the retrospective na ture of the study.

#### Participant enrollment and study design

In this multicenter, retrospective observational study, a general hospital database was used with the clinical information of all patients admitted to two tertiary hospitals (Center 1: the Fourth Medical Center of the Chinese PLA General Hospital; Center 2: the Sixth Medical Center of the Chinese PLA General Hospital). From 1 June 2021, to 31 May 2024, 1954 critically ill patients were admitted to the Emergency ICU (EICU) or Surgical ICU (SICU) in two medical institutions involved in this study (Figure 1). Adult patients who met the diagnostic criteria of sepsis 3.0 ^31^ were enrolled. The exclusion criteria of our study were as follows: (1) <18 years old; (2) repeated admission to the ICU; (3) ICU stay <24 hours (h); (4) anemia was present on admission; (5) history of diseases of the blood and coagulation system; (6) history of blood transfusion within one month prior to admission; (7) presence of surgical blood loss; (8) malignant tumors; (9) HIV-positive status; (10) pregnancy; and (11) no available information. It was confirmed that cases with the absence of more than three key indicators are regarded as having no available information and will be excluded.

The patients all received 24h medical monitoring and care provided by the staff and clinicians in EICU and SICU. The therapeutic strategy was guided following the instructions of Surviving Sepsis Campaign Guideline.^31^ Based upon available clinical information during treatment, enrolled patients were identified as the anemia-group or the non-anemia control group.

#### Definitions

Anemia is defined as a condition in which hemoglobin concentration and/or red blood cell (RBC) numbers are lower than normal and insufficient to meet an individual’s physiological needs. The diagnostic criteria for anemia adopted in this study were: female, hemoglobin <120 g/L; male, hemoglobin <130 g/L.^32^ Moreover, the degree of anemia is divided into three levels according to the hemoglobin concentration: mild, >100 g/L; moderate, 80-100 g/L; and severe, ≤80 g/L.^5^

#### Outcomes and follow-up

In our retrospective observational study, the outcomes were defined as the occurrence of anemia for ICU patients with sepsis during hospitalization. The start date of follow-up was considered as the date of the patient’s admission.

#### Data collection

We gathered the data from the electronic and paper medical records of patients, and data collection was conducted at the time of admission and before any blood transfusion. Data regarding demographics, vital signs, comorbidities, SOFA scores (on the admission day), APACHE II scores (on the admission day), laboratory results, and primary outcomes were recorded on a previously designed e-sheet.

In 44 of the 723 patients, important data such as PCT, IL-6, Alb, etc. were randomly missing, while prognostic data were not lacking and were therefore included in the analysis of outcomes. As the data missing rate < 10%, we did not perform data interpolation.

### Method details

#### Laboratory parameter assay

Blood samples for the blood routine tests and other laboratory parameter examination were obtained from venous or arterial puncture after presentation to the EICU and SICU, and were analyzed within 3 h. Importantly, we ensure that the collected data were obtained before the patient received blood transfusion treatment. The blood routine test was performed every day during hospitalization in order to monitor the hemoglobin levels.

The routine blood tests were performed by using an XN-10 (B4) automated hematology analyzer (Sysmex, Kobe, Japan). The Alb, ChE and creatinine were obtained by using the automatic biochemical analyzer (Roche, cobasc501), and the PCT and IL-6 were measured using the electrochemical luminescence immunoassay system (Roche, cobas e411). The CRP was examined by using the CRP quantitative analyzer (QuickRead go system). The lactate level was obtained through an arterial blood gas analyzer (Radiometer, ABL-90). The normal ranges for laboratory parameters in our study are as follows: white blood cells, 3.5-9.5×10^9^/L; platelets 125-350×10^9^/L; Alb, 35-50 g/L; ChE 5-12kU/L; creatinine 57-111μmol/L; PCT <0.05 ng/ml; IL-6 <7pg/ml; CRP <10mg/L; lactate 0.4-2.2mmol/L.

#### Development and validation of prediction model

The eligible sepsis patients (n=610) in Center 1 were randomly assigned to either the training set (n=427) or the internal validation set (n=183) at a 7:3 ratio. Additionally, 113 patients hospitalized at Center 2 were included in the external validation set. We developed the prediction model in the training set and verified its performance in the validation set. In the training set, univariate logistic regression analysis was used to screen the factors with *p* < 0.05, which were put into a multivariate model for stepwise regression to select some possible predictors. The data were compared by using odds ratios within 95% CI. These predictive factors were incorporated into a multivariable model to estimate the probability of anemia development in sepsis patients. A calibration curve, DCA and CIC were used to assess the predicting performance of prediction model in the training set and validation set.

### Quantification and statistical analysis

The target sample size and events per predictor were computed in accordance with the method described by Riley et al.^33^ The Shapiro-Wilk test was used to analyse the theoretical distribution of the continuous variables. For our study, mean ± standard deviation (Mean ± SD) and medians with interquartile ranges (IQR) were adopted to described the normally-distributed and nonnormally-distributed of measurement data, respectively. Categorical data are expressed as the number (percentage). Comparison of two groups was performed using chi-square test for categorical variables, as well as student t test or Mann-Whitney U tests for continuous variables. Comparisons of more than two groups were performed using one-way analysis of variance (ANOVA) followed by the least significant difference (LSD) multiple comparison test.

Lasso regression was performed to screen the independent parameters, and multivariate logistic regression analysis was used to screen the risk factors. The AUC and the Hosmer-Lemeshow goodness of fit test were adopted to evaluate the predicting performance of constructed prediction model. All statistical analyses were performed with SPSS® (version 22.0, Chicago, USA) and MedCalc® (version 11.4.40, Belgium). In addition, the sample size calculation and nomogram prediction model was constructed by using R software (version 4.5.1, Vienna, Austria) pmsampsize and rms package. *p* < 0.05 was considered to be statistically significant.
