# Supplementary material for: Nomogram for anemia risk prediction and validation in sepsis patients
Source: iScience. 2025 Dec 18;29(1):114483. doi: 10.1016/j.isci.2025.114483 (PMC12804608; doi:10.1016/j.isci.2025.114483)
Supplement: Document S1. Tables S1 and S2 [file mmc1.pdf]

iScience, Volume *29*

Supplemental information

Nomogram for anemia risk prediction

and validation in sepsis patients

Songwei Li, Yahui Gao, Tianyu Xin, Jianying Guo, Liwei Pang, Li Yan, Fei She, Xiaohong Li, and Shuangqing Liu

Supplementary Table S1. Comparison of severity scores between two groups in the training set

**severity scores**	**Group**		*t/Z*	*p* value
	Anemia n=290	Non-anemia n=137		
APACHE II	10.30±2.72	8.71±2.25	5.941	<0.001
SOFA	5 (4-7)	5 (4-6)	-0.876	0.381

Supplementary Table S2. Comparison of biological indicators between two groups in the training set.

biomarkers	Group				
	Anemia n=290				Non-anemia n=137
	Total	Mild n=113	Moderate n=100	Severe n=77	
Alb (g/L)	31.61±4.81***	33.39±4.48	31.93±4.37△	28.58±4.42▲▲▲	37.44±4.11
PCT (ng/ml)	7.23±3.11**	7.02±3.01	7.65±3.38	7.51±3.07	6.29±2.07

Compared with the non-anemia group, ****p* < 0.001, ***p* < 0.01. In the anemia group, compared with the mild anemia subgroup, △*p* <0.05; compared with the moderate anemia subgroup, ▲▲▲*p* <0.001.
